# AMPA Receptor Regulation at the mRNA and Protein Level in Rat Primary Cortical Cultures

**DOI:** 10.1371/journal.pone.0025350

**Published:** 2011-09-22

**Authors:** Cesare Orlandi, Luca La Via, Daniela Bonini, Cristina Mora, Isabella Russo, Alessandro Barbon, Sergio Barlati

**Affiliations:** Division of Biology and Genetics, Department of Biomedical Sciences and Biotechnologies and National Institute of Neuroscience, University of Brescia, Brescia, Italy; Lehigh University, United States of America

## Abstract

Ionotropic glutamate α-amino-3-hydroxy-5-methyl-4-isoxazole propionic acid (AMPA) receptors are the major mediators of fast synaptic neurotransmission. In this work, we used primary cortical cultures from rats as a model system to study AMPA receptor regulation during *in vitro* cell maturation and after synaptic activity modifications. The levels of AMPA receptor mRNA and protein, along with the alternative splicing and RNA editing of the AMPA receptor subunit (GluR1-4) mRNAs, were analyzed in immature (DIV5) and mature (DIV26) rat neuronal cultures. We observed an increase in the expression of all four AMPA receptor subunits during *in vitro* neuronal maturation. This finding might be due to the formation of new synapses between neurons during the development of a complex neuronal network. We also analyzed the effects of stimulation (KCl and glutamate) and inhibition (APV/TTX) on rat mature neuronal cultures (DIV26): stimulation with KCl led to an overall down-regulation of GluR1 and GluR3 AMPA receptor subunits and an up-regulation of the GluR2 subunit. Similarly, glutamate treatment induced a significant down-regulation of GluR1 together with an up-regulation of GluR2. In contrast, the chronic blockade of neuronal activity that resulted from APV/TTX treatment up-regulated GluR1 and GluR3 with a parallel down-regulation of GluR2 and GluR4. RNA editing at the R/G site increased during neuronal cell maturation for all AMPA receptors (from 8–39% at DIV5 to 28–67% at DIV26). Unexpectedly, all the treatments tested induced a marked reduction (ranging from −9% to −52%) of R/G editing levels in mature neurons, primarily for the mRNA flip variant.

In summary, we showed that cultured rat cortical neurons are able to vary the stoichiometric ratios of the AMPA receptor subunits and to control post-transcriptional processes to adapt fast synaptic transmission under different environmental conditions.

## Introduction

The ionotropic glutamate α-amino-3-hydroxy-5-methyl-4-isoxazole propionic acid (AMPA) receptors are important mediators of fast synaptic neurotransmission *in vivo* and in cultured cortical neurons. The number, composition and localization, as much as post-transcriptional and translational regulation, of AMPA receptors in neurons are critically important factors that determine the neuronal response to glutamate and play a significant role in synaptic plasticity [1], [2]. AMPA receptors have been implicated in several neurodevelopmental disorders including Fragile-X mental retardation [3] and schizophrenia [4], as well as in neurodegenerative conditions such as Alzheimer's disease [5], motor neuron disease (ALS) [6], [7], stroke [7], [8] and Parkinson's disease [5], [8]. AMPA receptors also contribute to the proliferation of glioblastoma tumors [9], [10] and have been suggested to have a role in depression [5], [8] and in the seizure spread and neuronal damage associated with epilepsy [11]. For a review, see Bowie (2008) [12].

Activity-dependent regulation of synaptic AMPA receptor number and composition has a critical role in long-lasting synaptic plasticity. The regulated trafficking of postsynaptic AMPA receptors has been shown to be an important mechanism underlying activity-induced changes in synaptic transmission [13], [14], [15]. Moreover, post-transcriptional modifications of AMPA receptor mRNAs such as RNA editing and alternative splicing are functionally relevant in the regulation of glutamatergic neurotransmission. Indeed, they represent a sophisticated mechanism that allows to modify the receptor subunits in positions that are key in regulating the permeability of the channel, the kinetics of pore opening, the time of desensitization of the receptor, as well as the time of recovery from desensitization [16], [17], [18].

The RNA editing process catalyzed by adenosine deaminases that act on RNA (ADARs) 1 and 2 [19] alters one or more of the translation codons, giving rise to functionally distinct proteins from a single gene. GluR2, GluR3 and GluR4 are edited at different sites that have been characterized by the amino acid substitution that occurs; these include the Q/R site in AMPA receptor GluR2 and the R/G site in GluR2, GluR3 and GluR4 [16], [17], [18]. GluR2 Q/R-edited subunits give the channels a linear current-voltage relationship [20] and impermeability to Ca^2+^. On the other hand, R/G editing modulates the kinetic properties of AMPA receptor channels [21], thus determining the time course of desensitization and resensitization [21], [22].

Alternative splicing of the flip/flop cassette is another post-transcriptional modification that can alter AMPA receptor function. It consists of the insertion of a mutually exclusive exon into the mature mRNA of all 4 AMPA receptor subunits that results in the insertion of 38 amino acids into the extracellular loop prior to the 4^th^ transmembrane domain [23]. This modification generates channels that differ in their kinetic properties, including the time course of desensitization and resensitization [24], [25], [26].

Interestingly, the R/G editing site is located one nucleotide upstream of the sequences involved in the splicing events forming the flip/flop isoforms and it seems to affect both the desensitization properties of the AMPA receptor channels [21], [22] and splicing [21], [27], even though the functional correlation between the two post-transcriptional events has not yet been completely elucidated [21], [28].

In attempts to further understand the functional features of AMPA receptors, animal and cell culture model systems have been used extensively [29], [30], [31]. Primary neuronal cultures have been shown to be a powerful tool for studying the cellular and molecular mechanisms of neuronal development and for studying neuronal plasticity under different kinds of treatments capable of modulating synaptic activity [29], [30]. Cortical neurons grown for a few days *in vitro* seem to mimic the first stages of central nervous system development [32] and are thought to represent immature neurons [33]. After 3 to 4 weeks, cultured neurons give rise to a complex network, forming synapses with other cells [34]. In such cultures, AMPA receptor subunits exhibit cell type-specific expression. Whereas a wide variety of neurons express GluR1 and GluR2, only about 20% of the cells in cultured neuronal populations express significant levels of GluR3 or GluR4 [35]. However, little is known about the post-transcriptional regulation of AMPA receptor mRNAs, such as RNA editing and splicing, that occurs during neuronal maturation and in response to modulation of neuronal activity.

Accordingly to the growing recognition of the importance of AMPA receptor regulation during neuronal development and in adult brain disorders, we performed a comprehensive characterization of AMPA receptor protein levels, mRNA expression and, especially, post-transcriptional regulation over the course of four weeks of *in vitro* maturation in cortical neuronal cultures. We also evaluated the effects of two different paradigms of neuronal activity stimulation (KCl and glutamate treatments), as well as blockade (APV/TTX), on the level and post-transcriptional regulation of AMPA receptors.

## Results

### Characterization of rat primary cortical cultures by immunofluorescence

**Figure 1:**
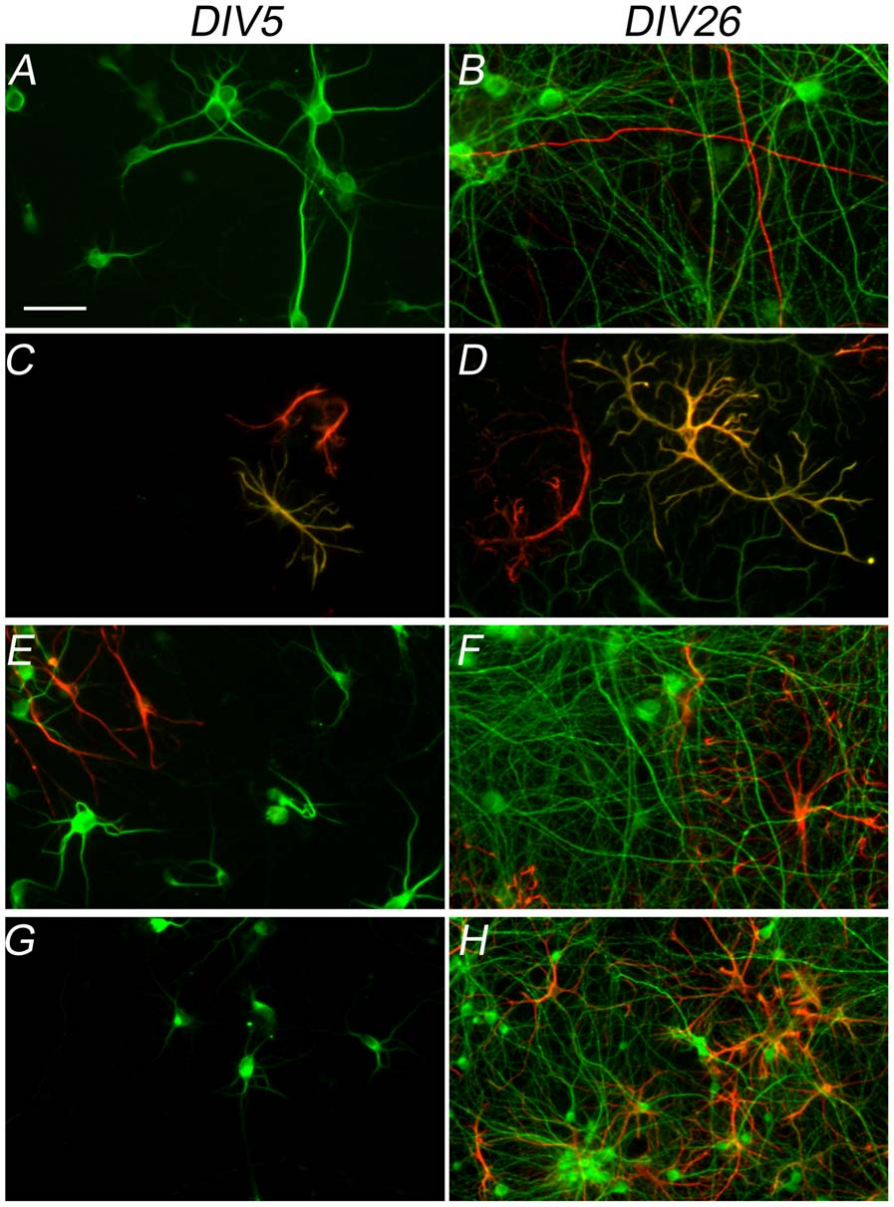
Immunofluorescence analysis of rat cortical cultures. Double labeling of rat cortical neurons, shown at 20× magnification. The left column shows the immunolabeling of immature cultures at DIV5, while the right column shows the localization of the same markers in mature cultures at DIV26. (A, B) Double labeling of dendrites with antibodies to MAP2 (green) and of axons with antibodies to neurofilament heavy chain subunit (≥200 KDa; red). (C, D) Double labeling of the glial population with antibodies to GFAP (green) and of neuronal precursor cells with antibodies to nestin (red). (E, F) Double labeling of dendrites with anti-MAP2 (green) and of neuronal precursor cells with anti-nestin (red). (G and H) Double labeling of dendrites with anti-MAP2 (green) and of glial cells with anti-GFAP (red). Scale bar: 50 µm.

Immunofluorescence analyses were carried out to characterize the rat primary cortical cultures (Fig. 1). Primary antibodies against microtubule-associated protein 2 (MAP2) and the heavy chain (≥200 KDa) of neurofilament (NF-200) were used to assess neural maturation. In immature cultures (DIV5), the neurites showed only MAP2 immunoreactivity, while in mature cultures (DIV26), an extremely branched dendritic tree with well-defined dendrites and axons that exhibited immunoreactivity to NF-200 was observed (Fig. 1A and B). To test for the presence of non-neuronal cells in these cultures, double immunolabeling for glial fibrillary acidic protein (GFAP) and nestin was performed (Fig. 1C and D). At DIV5, less than 1% of the cells were GFAP-positive and 10–20% were nestin-positive, indicating the presence of precursor cells with the ability to differentiate into glial or neuronal cells. At DIV26, the population of nestin-positive cells increased and cell differentiation was primarily biased toward glial cells, as demonstrated by the colocalization of GFAP and nestin signals (Fig. 1D). Double immunolabeling for MAP2 and nestin was used to determine whether differentiation of nestin-positive precursor cells into neuronal cells resulted in an increased number of neurons in the cultures. Colocalization of these antigens, which would have confirmed the differentiation of nestin-expressing precursors into neuronal cells, was not found (Fig. 1E and F). Finally, double staining for MAP2 and GFAP was used to evaluate the glial population present in rat cortical cultures (Fig. 1G and H). Immature cultures had less than 1% glial cells, while in the 26-day cultures, approximately 20% of the cells were glial cells likely deriving from the differentiation of nestin-positive precursors as already reported [32], [36].

### Analysis of the levels of AMPA receptor subunits during cell maturation

**Figure 2:**
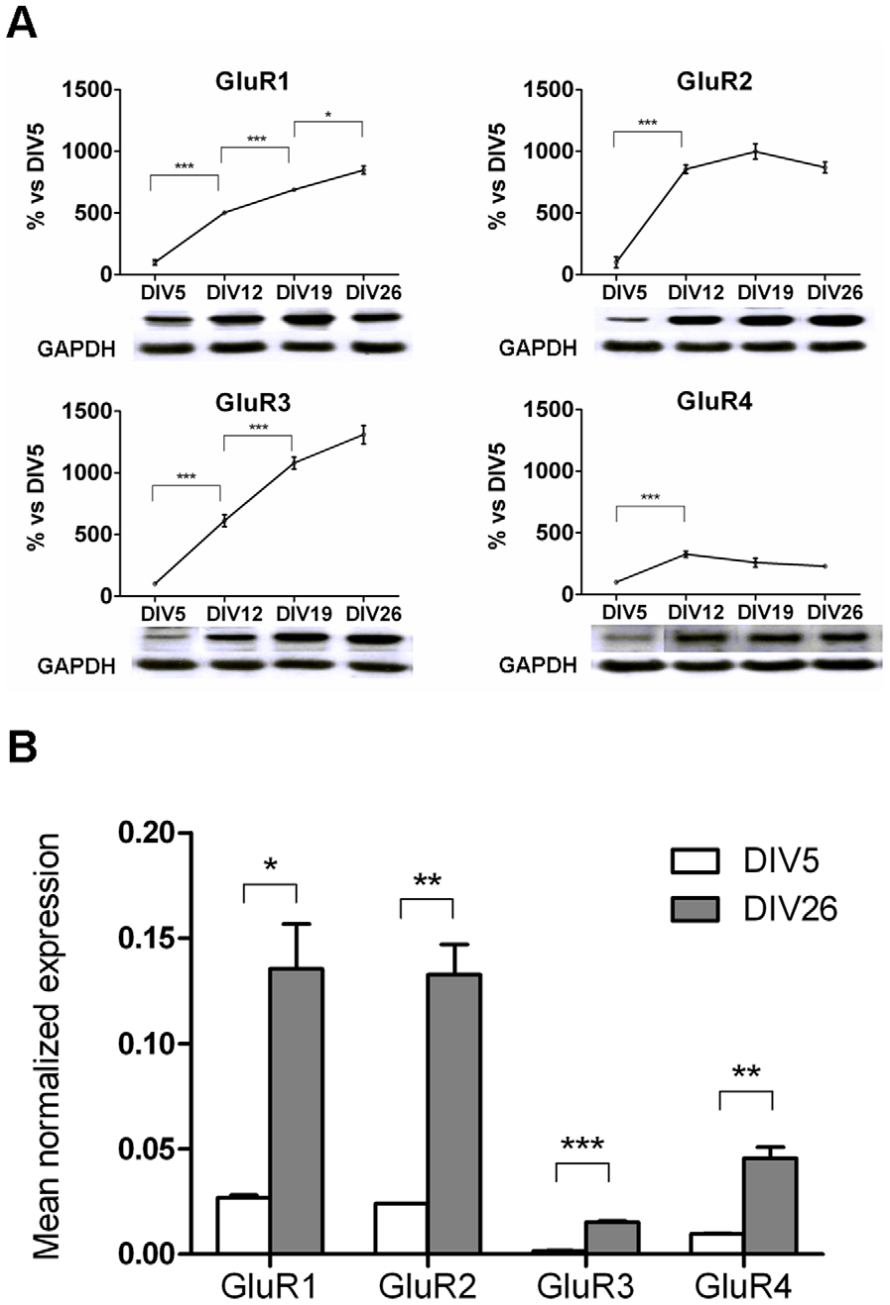
mRNA and protein levels of AMPA receptor subunits during cell maturation. (A) Western blot analysis of the four AMPA receptor subunits. A time course at four different stages of maturation of cultured neurons is shown. DIV5 protein levels of AMPA receptor subunits were set to 100%. GAPDH was used as loading control and the results are the mean ± S.E. of 3 independent experiments, performed in independent preparations. Statistical analysis was performed by one-way ANOVA followed by the Bonferroni's multiple comparison test (*p<0.05, **p<0.01, ***p<0.001). (B) Normalized expression level of the four AMPA receptor subunit mRNAs analyzed by qGene software [61] in immature (DIV5) and mature (DIV26) cortical cultures. Each value is normalized to a geometric mean of three house-keeping genes: GAPDH, RPLR2 and β-actin. The results are the mean ± S.E. of 3 independent experiments, performed in independent preparations. Statistical analysis was done using Student's *t* test.

The protein levels of AMPA receptor subunits in the cultures were evaluated by western blot analysis at four time points (DIV5, DIV12, DIV19 and DIV 26). With time in culture, an increase of the protein levels for all four AMPA receptor subunits was observed. The transition from DIV5 to DIV12 involved the most significant (ten-fold) increase for GluR1, GluR2 and GluR3, while the GluR4 subunit, primarily expressed at the embryonic stages *in vivo*, increased only two- to three-fold (Fig. 2A).

Quantitative real-time PCR confirmed the increased expression of the four AMPA receptor subunits from DIV5 to DIV26 and showed that GluR1 and GluR2 are the most highly expressed subunits at each stage of development; their expression is approximately nine-fold higher than that of GluR3 and three-fold higher than that of GluR4 (Fig. 2B). A good correlation was therefore observed between mRNA and protein levels during cell maturation.

### AMPA receptor subunits are predominantly expressed in neuronal cells

**Figure 3:**
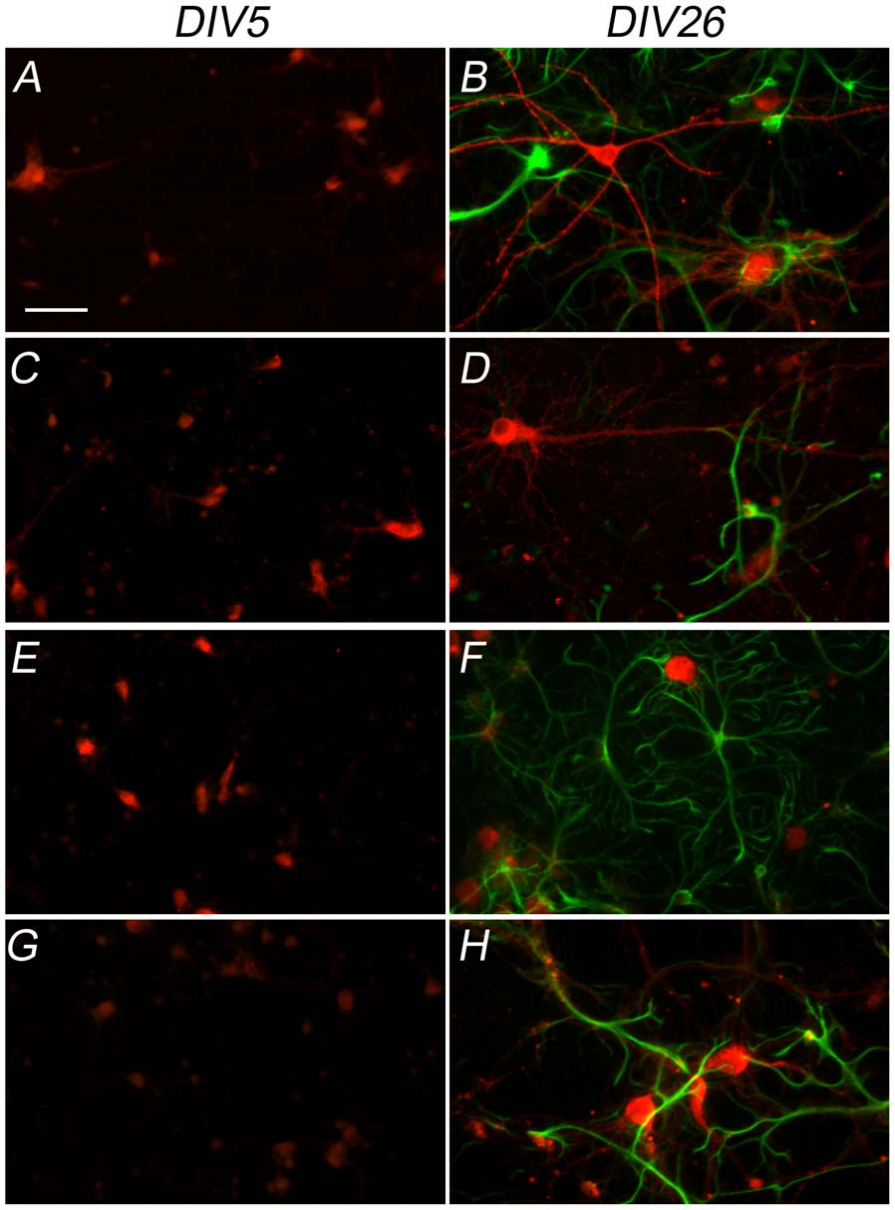
AMPA receptor expression is predominant in neuronal cells. Double labeling of rat cortical neurons at DIV5 and DIV26 (20×) with an antibody against the glial marker GFAP (green) and with antibodies against the four AMPA receptor subunits: (A, B) GluR1; (C, D) GluR2; (E, F) GluR3 and (G, H) GluR4 (red). The images were acquired from rare regions with high density of glial cells to show that the GFAP-positive cells do not express AMPA receptors. Scale bar: 50 µm.

Immunofluorescence analyses were carried out in order to establish in the rat cortical cultures the cellular populations expressing AMPA receptor subunits (Fig. 3). Immature cultures (DIV5) showed very few GFAP-positive cells and low AMPA receptors expression. Cortical cultures at DIV26 showed that all four AMPA receptor subunits were present in the neuronal population, while only a small degree of colocalization of AMPA receptor subunits with the glial marker GFAP was observed. These data indicate that the glial population doesn't contribute significantly to the total amount of AMPA receptors analyzed with western blot and real-time PCR.

### GluR1-4 flip and flop isoform relative expression and GluR2-4 R/G site editing during cell maturation

**Figure 4:**
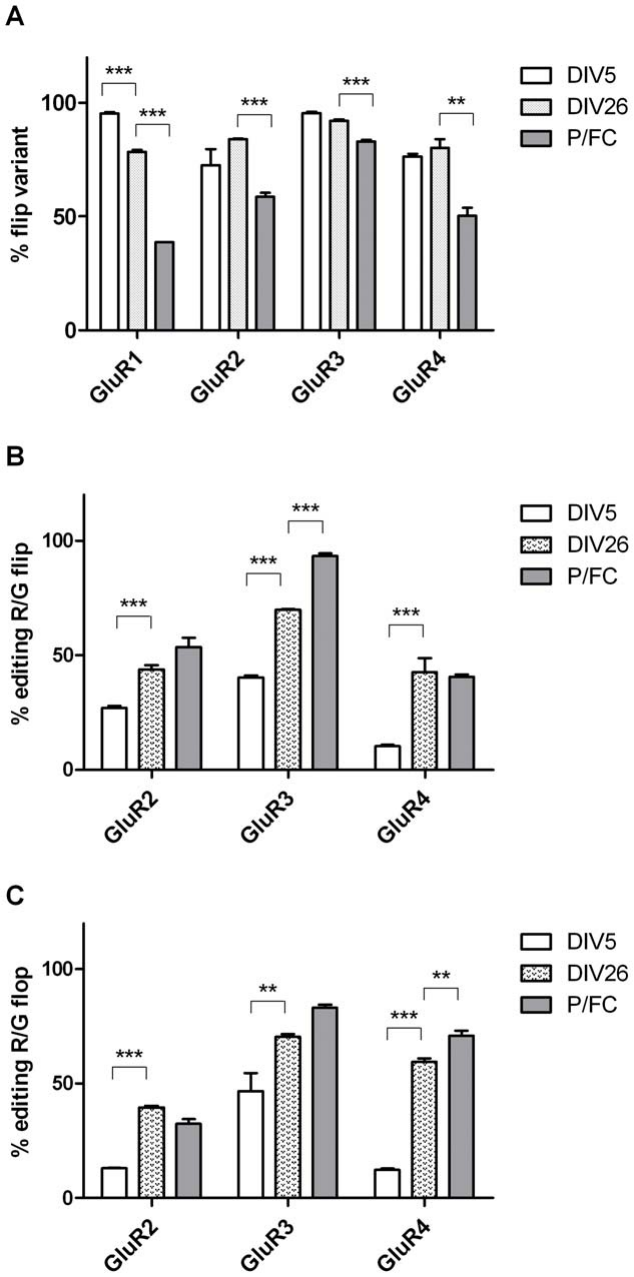
GluR1-4 flip and flop isoform relative expression and GluR2-4 R/G site editing during cell maturation. (A) The graphs represent the percentage of the flip isoform among the total flip + flop isoforms present during the maturation of cortical cultured neurons and in extracts of adult rat prefrontal/frontal cortex (P/FC). (B, C) Editing levels at the R/G site of the flip (B) and flop (C) variants of subunit GluR2-4 mRNAs. The results are the mean ± S.E. of 3 independent experiments, performed in independent preparations. Statistical analysis was performed by one-way ANOVA followed by the Bonferroni's multiple comparison test (*p<0.05, **p<0.01, ***p<0.001).

The relative expressions of the flip/flop isoforms of GluR subunit mRNAs were evaluated by sequence analysis. In immature neurons, the flip variants were the predominant isoforms (95% for GluR1 and GluR3, 73% for GluR2 and 76% for GluR4). Following *in vitro* maturation, a slight increase in the GluR1 flop isoform at the expense of flip was found, while the GluR2-4 splice variants were unchanged (Fig. 4A). Nevertheless, the flip variant remained the predominant isoform in mature neurons (78% for GluR1, 84% for GluR2, 92% for GluR3 and 80% for GluR4). It is worth noting that in the adult rat brain, the relative expression levels of the flip isoform (38% for GluR1, 58% for GluR2, 82% for GluR3 and 40% for GluR4) are lower than those in neuronal cortical cultures.

Editing levels of the AMPA receptor subunits at R/G sites varied considerably during cell maturation (Fig. 4B). Editing at the R/G site of the GluR2 and GluR3 subunits increased gradually from DIV5 to DIV26 for both the flip and the flop isoforms (GluR2-Flip DIV5: 27%, DIV26: 44%; GluR2-Flop DIV5: 13%, DIV26: 40%; GluR3-Flip DIV5: 40%, DIV26: 70%; GluR3-Flop DIV5: 47%, DIV26: 70%). For GluR4 transcripts, the editing levels at the R/G site of both flip and flop isoforms were approximately 10% at DIV5; by DIV26, they had increased to different levels for the two isoforms (GluR4-flip 42% and GluR4-flop 60%). As shown in Figure 4, the R/G editing levels in adult brain are only slightly higher than those observed at DIV26, confirming that an *in vitro* maturation process comparable to that seen *in vivo* occurs.

### Analysis of the levels of AMPA receptor subunits after acute and chronic treatment with KCl and APV/TTX

**Figure 5:**
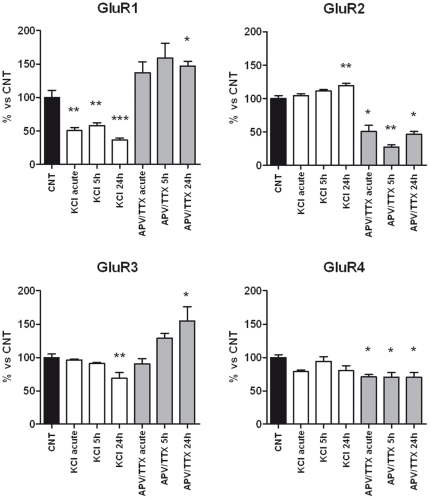
Protein levels of AMPA receptor subunits after acute and chronic treatment with KCl and APV/TTX. GluR1-4 protein levels in primary cortical cultures (DIV26) after both acute and chronic treatment with KCl and APV/TTX. The results are expressed as the mean ± S.E. of 3 independent experiments performed in independent preparations and reported as percentage of non-treated control (CNT). Statistical analysis was performed by one-way ANOVA followed by the Dunnett test (*p<0.05, **p<0.01, ***p<0.001).

We evaluated the effects of neuronal activity at the protein level and post-transcriptional regulation of AMPA receptors. Western blot analysis showed a great influence of KCl depolarization and APV/TTX blockade on the level of AMPA receptor subunits (Fig. 5). In mature cultures (DIV26), both acute and chronic stimulation of synapses led to an overall down-regulation of GluR1 (−42% to −63%) and GluR3 (−31% at 24 h), while they induced an increase in GluR2 (+20% at 24 h). Conversely, chronic blockade of synaptic activity up-regulated GluR1 (+47% at 24 h) and GluR3 (+55% at 24 h) with a parallel down-regulation of GluR2 (−49% to −72%) and GluR4 (−29% to −30%).

### Effects of treatment on GluR1-4 splicing and editing

**Figure 6:**
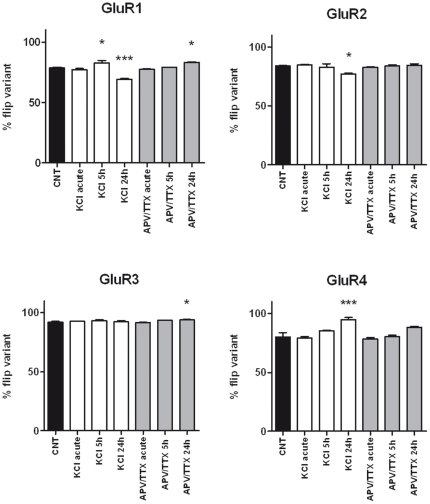
GluR1-4 splicing levels after acute and chronic treatment with KCl and APV/TTX. Relative expression of the flip variant as a percentage of the total flip + flop for each of the four AMPA receptor subunits. The results are expressed as the mean ± S.E. of 3 independent experiments performed in independent preparations. Statistical analysis was performed by one-way ANOVA followed by the Dunnett test (*p<0.05, **p<0.01, ***p<0.001).

**Figure 7:**
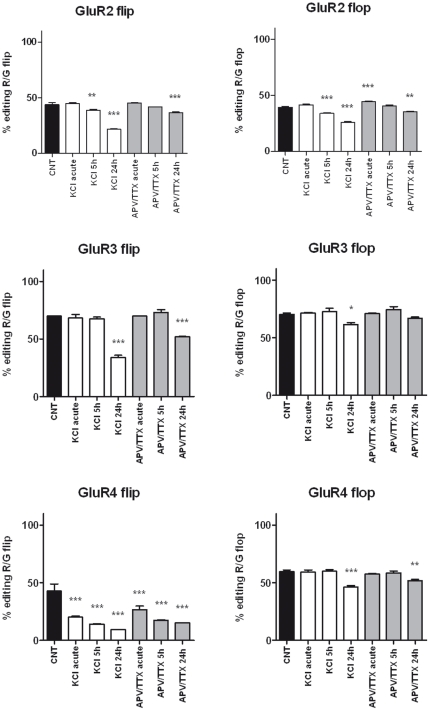
GluR2-4 R/G editing levels after acute and chronic treatment with KCl and APV/TTX. Editing levels at the R/G site of subunits GluR2-4 for the flip and flop variants. The effects of KCl and APV/TTX treatments on the editing levels at the R/G site are shown separately for each variant. The results are expressed as the mean ± S.E. of 3 independent experiments performed in independent preparations. Statistical analysis was performed by one-way ANOVA followed by the Dunnett test (*p<0.05, **p<0.01, ***p<0.001).

A 24-hour treatment with either KCl or APV/TTX influenced alternative splicing in mature cultures (Fig. 6). KCl depolarization reduced the flip expression of GluR1 (−9%) and GluR2 (−7%) and increased the expression of the GluR4 flip isoform (+14%). APV/TTX treatment, on the other hand, generated a mild but significant increase in the relative flip expression for GluR1 (+5%) and GluR3 (+2%) only. A more substantial decrease in editing at the R/G site of each AMPA receptor subunit mRNA was observed after chronic treatment with KCl or APV/TTX (Fig. 7). In mature neurons, chronic KCl depolarization markedly reduced editing of the flip isoform at this site (−22% for GluR2, −36% for GluR3 and −33% for GluR4) with a lower decrease in the editing of the flop isoform (−14% for GluR2, −9% for GluR3 and −13% for GluR4). Chronic treatment of mature neurons with APV/TTX led to a similar general reduction (−7% for GluR2, −18% for GluR3 and −27% for GluR4) in flip isoform editing, with a smaller effect on flop isoform editing (−4% for GluR2 and −8% for GluR4).

Both in primary cortical cultures and in extracts from adult rat brains, the level of editing at the Q/R site of GluR2 was always 100%. None of the treatments used had an effect on the Q/R site (data not shown).

### Effects of glutamate treatment on GluR subunit expression, splicing and editing

**Figure 8:**
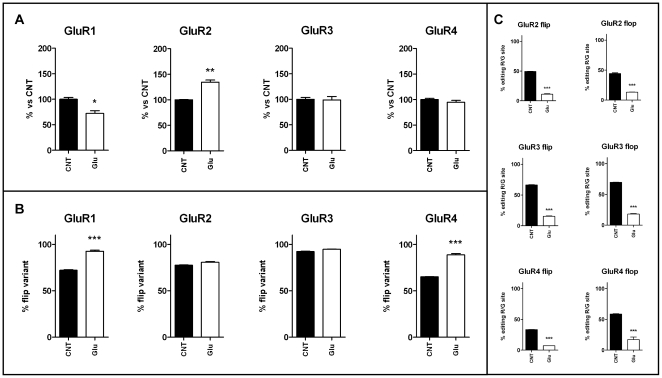
Protein levels, flip/flop splicing and RNA editing at the R/G site of GluR1-4 after 24-hour treatment with glutamate. (A) Western blot of the four AMPA receptor subunits after 24-hour treatment with glutamate on mature cortical cultures. The results are expressed as the mean ± S.E. of 3 independent experiments performed in independent preparations and reported as percentage of non-treated control (CNT). (B) The graphs represent the percentage of the flip isoform among the total flip + flop isoforms after glutamate treatment. (C) Editing levels at the R/G site of subunits GluR2-4 for the flip and flop variants. The results are expressed as the mean ± S.E. of 3 independent experiments performed in independent preparations. Statistical analysis was performed by unpaired Student's t-tests (*p<0.05, **p<0.01, ***p<0.001).

A 24-hour treatment with glutamate on mature cortical cultures induced changes in expression levels of the different AMPA receptor subunits that are comparable with those observed with KCl treatments (Fig. 8A). Western blot analyses of the four AMPA receptor subunits revealed a decrease of GluR1 (−28%) and an increased of GluR2 (+34%) while GluR3 and GluR4 levels were not changed. Concerning the expression of the flip and flop splicing isoforms, glutamate treatment produced a significant increase in the relative expression of the flip isoform of GluR1 and GluR4 (+20.4% for GluR1 and +23.6% for GluR4) (Fig. 8B). Finally, the analysis of the RNA editing levels at the R/G site showed a marked reduction for all the four AMPA receptor subunits both for the flip isoform (−38.3% for GluR2, −50.9% for GluR3 and −26.1% for GluR4) and for the flop isoform (−31.0% for GluR2, −51.6% for GluR3 and −41.4% for GluR4) (Fig. 8C). No effect has been observed on GluR2 Q/R editing level. These data are consistent with those observed after KCl depolarizing treatments.

## Discussion

In the present work, we used an *in vitro* model of cortical neuron maturation to investigate the relationship between neuronal activity and the levels and post-transcriptional regulation of glutamate AMPA receptor subunits by performing a quantitative molecular analysis of the four AMPA receptor subunits (GluR1 and GluR2, and also the less known GluR3 and GluR4) and a thorough analysis of post-transcriptional events such as flip/flop alternative splicing in association with RNA-editing. This new bulk of data represents a step toward an increased knowledge of the fine-tuning mechanisms regulating glutamatergic neurotransmission.

We observed that *in vitro* neuronal maturation led to increased expression of all four AMPA receptor subunits both at the mRNA and protein levels. This might be due to the formation of new synapses between neurons during the development of a complex neuronal network. In primary cortical cultures, AMPA receptors are mainly expressed by neuronal population, while the expression by glial cells is negligible. Thus, the evaluation of receptor expression patterns, splice variants and RNA editing carried out in this work reflects the characteristics of receptors located primarily in neuronal cells. Concerning editing analysis, the Q/R site of GluR2 mRNA was fully edited in the cortical cultures at all time points studied, similar to results with adult rat brains [37] and other neuronal cultures [38]. The GluR2 Q/R-edited subunit is the most relevant for determining the conductance properties of AMPA receptors [1]. GluR2 Q/R-edited subunits give the channels a linear current-voltage relationship [20] and impermeability to Ca^2+^; AMPA receptors that lack GluR2 are permeable to Ca^2+^ [39]. On the other hand, in the embryonic brain the extent of R/G editing is generally low for both flip/flop splice forms, but it increases dramatically during development [21]. Our *in vitro* results, in agreement with *in vivo* data, show an increase in the R/G editing levels of GluR2-4 AMPA receptors with time in culture, with an amplitude that is isoform specific, that might results in alterations in the intrinsic kinetic properties of the receptor [21]. The main function of R/G editing is to modulate the kinetic properties of AMPA receptor channels in association with alternative splicing at the flip/flop cassette [21], thus determining the time course for desensitization and resensitization [21], [22]. Indeed, AMPA receptors coded for by the flip version of the mRNA take longer to desensitize than those coded for by the flop form [25], [26]. Moreover, AMPA receptors that contain an edited (G) subunit, show a faster recovery rate from desensitization than those with an unedited (R) form [21], [22]. The increased editing at the R/G site observed during neuron maturation could accelerate the kinetic of resensitization of AMPA receptors, thus increasing the response to external stimuli. We further showed that, in neuronal cultures, there is predominant expression of flip splicing variants, while in the adult brain the ratio between the two isoforms is almost equal, as already reported [23], [40].

In addition to demonstrating physiological modifications of GluR levels during neuronal maturation, our data showed that modulation of neuronal activity in rat primary cortical cultures can modify the properties of glutamate AMPA receptors by changing receptor protein expression and regulating simultaneously post-transcriptional processes.

Glutamate and KCl treatments induced a down-regulation of the amount of GluR1, with a concomitant up-regulation of GluR2 protein, while an opposite effect was observed after APV/TTX treatment, after which GluR1 subunit was up-regulated and GluR2 down-regulated. The less expressed subunits GluR3 and GluR4 were not affected by glutamate treatment, while they showed peculiar variations after chronic KCl and APV/TTX treatments. These data show that treatments that chronically induce neuronal activation lead to increased number of GluR2-containing receptors; this might lead to the presence of more Ca^2+^-impermeable AMPA channels. Moreover, besides being impermeable to Ca^2+^, GluR2 subunit is mainly in the flip version and it has a longer recovery time from desensitization [24]. AMPA channels containing the GluR2 subunit would be less efficient in responding to a train of pulses. Accordingly, GluR2 levels are tightly controlled by transcriptional regulation [41] and also by proteasome degradation [42], [43]; it has been shown that a loss of regulation of the GluR2 subunit contributes to the excitotoxicity mediated by the expression of Ca^2+^-permeable AMPA receptors during cerebral ischemia [44], [45].

On the other hand, we showed that chronic blocking treatment with APV/TTX leads to an increase in GluR1 and GluR3 subunit level. The parallel down-regulation of GluR2 and up-regulation of GluR1 and GluR3 would produce an imbalance in the ratio between the AMPA receptor subunits that might favor Ca^2+^-permeable channels. The mechanisms responsible for the changes in the GluR levels after these paradigms of neuronal activity modulation remain to be established. The origin of these changes might reside in the transcriptional/translational modulation or in the mechanism of proteasome-mediated degradation of the receptors, a process known to be regulated by synaptic activity [42], [43].

Concerning the analysis of post-transcriptional modifications, the manipulation of neuronal activity did not alter GluR2 Q/R editing levels, and conceivably no modification in the permeability of GluR2 containing-AMPA receptors should be present. A recent work evidenced a reduction in Q/R editing after a 4-hour treatment with 100 µM glutamate [46] and this down-regulation was directly linked to glutamate excitotoxicity. The lower concentration used in our study does not induce the same down-regulation of Q/R editing level and it might have no effect on cell survival. In addition we showed that KCl, glutamate and also APV/TTX treatments modulate R/G editing and splicing. Each AMPA receptor subunit (except GluR1 that is not edited) exists in four different mRNA isoforms deriving from the sum of flip/flop splicing process and editing at R/G site. Chronic treatments that triggered neuronal activity, such as KCl and glutamate, induced a significant down-regulation of R/G editing. Especially, glutamate treatment has shown the strongest effect on all the AMPAR subunit R/G sites. It should be taken in account that also APV/TTX treatment, though to a lesser extent, induced a partial down-regulation of R/G editing; we cannot rule out that this unpredictable result could be an indirect effect of neuronal culture treatment. Additional work is needed to elucidate the correlation between APV/TTX treatment and RNA editing modulation.

In addition to changes in RNA editing levels, we found alterations also in the splicing pattern of the flip/flop exon cassette for AMPA receptor mRNAs after treatments. After glutamate treatment an increase of the flip form could be clearly detected. Isoform specific alterations, have also been observed with KCl and APV/TTX treatments that might be due to their indirect effect on AMPA channels.

In summary, for GluR2-4, both KCl and glutamate treatments induced an up-regulation of the R/G-unedited flip isoforms (A-flip), characterized by a decreased desensitization time constant (τ_D_) and an increased recovery time constant (τ_rec_) [1], [21], [24], in association with a concomitant decrease in the G-flip isoform. Surprisingly, a similar effect has been observed after APV/TTX treatment, though to a lesser extent (Fig. S1). These data indicate that modulation of editing and splicing cannot be directly linked to neuronal excitation or inhibition but that more complex mechanisms might take place to fine-tune AMPA receptor channel properties; moreover a clear correlation between editing and splicing processes has not yet been well-characterized [28]. Concerning GluR1, slight modifications in flip/flop relative expression were reported, although the two isoforms do not show marked differences in their kinetic properties [1], [47] so that these variations appeared to be functionally irrelevant for the channel.

Additional experiments are needed to clarify the cascade of events between the modulation of neuronal activity and the post-transcriptional modification of AMPA receptor subunits. This could represent a fine-tuning regulation of AMPA receptor properties in addition to the main effect observed on the protein amount of each subunit. The global effect of the molecular regulations analyzed should be responsible for the already reported changes in electrophysiological properties of glutamate AMPA receptors subsequent to neuronal activity modulation [48].

In conclusion, our results illustrate the relevance of tightly controlled expression and post-transcriptional modification of AMPA receptor subunits. We show that cultured rat cortical neurons are able to vary the stoichiometric ratios of the AMPA receptor subunits and to control editing and splicing processes to adapt fast synaptic transmission under different environmental conditions. Improved knowledge of the fine-tuning mechanisms underlying AMPA receptor-mediated neurotransmission in neuronal cultures will be useful in understanding the features of neuronal responses in both normal and pathological conditions.

## Materials and Methods

### Primary neuronal cell cultures and treatments

Our experiments complied with guidelines for the use of experimental animals issued by the European Community Council Directive 86/609/EEC and were approved by the Italian Ministry of Health (Project ID: 320/2010). Rat cortical cultures were prepared as previously described [32], [49]. In brief, cerebral cortices from day 18 Sprague-Dawley rat embryos (E18, Charles River Laboratories Inc., Wilmington, MA, USA) were mechanically dissociated by trituration in cold HBSS (Invitrogen, Carlsbad, CA, USA) containing 10 mM HEPES (pH 7.4). The suspension was allowed to settle for 5 min, and the top fraction, which contained more than 95% single cells, was collected. The neurons were centrifuged for 5 min at 200 g and resuspended in serum-free Neurobasal medium (Invitrogen) supplemented with B27 (Invitrogen), 30 U/ml penicillin (Sigma-Aldrich, St. Louis, MO, USA), 30 µg/ml streptomycin (Sigma-Aldrich) and 0.5 mM Glutamax (Invitrogen). Neurons were plated at a density of 30,000 cells/cm^2^ on poly-D-lysine- (Sigma-Aldrich) coated Petri dishes [33], [50]. In cultures plated at densities lower than 10,000 cells/cm^2^, neurons tended to remain in isolation. We used the higher cell density to encourage growing dendrites to quickly make contact with the axons of neighboring cells and thus form a dense network of interconnected processes. Three days after plating, 50% of the medium was replaced with fresh medium. Subsequently, half of the medium was replaced every 6 days for a maximum of 4 weeks.

Each experiment was performed using 3 independent preparations of DIV26 neurons as they express high levels of AMPA receptors and presumably present a greater number of active synapses. Acute treatments were carried out by adding 90 mM KCl to the culture medium in three 1-min applications over a 5-minute period [51] and then incubating the cells at 37°C for another 15 min. The chronic treatments consisted of adding 25 mM KCl to the cultures for 5 h or 24 h [52], [53], [54], [55]. Activity-blocking treatments consisted of adding 1 µM tetrodotoxin (TTX), a selective inhibitor of voltage-gated sodium channels, (Alomone Labs Ltd., Jerusalem, Israel) and 100 µM (2R)-amino-5-phosphonovaleric acid (APV), an NMDA receptors inhibitor (Sigma-Aldrich), to the culture medium for 5 min followed by a 15 min incubation at 37°C. In the chronic treatment condition, APV/TTX was added for 5 h or for 24 h [48], [51], [56], [57], [58]. Glutamate treatment was performed for 24 hours at a concentration of 50 µM [59], [60].

### Immunofluorescence

Rat cortical cultures were analyzed with immunofluorescence in order to determine the expression levels of several markers of neuronal maturation and the different cell types present in the cultures. We also tested the distribution of AMPA receptor subunits in neuronal and glial cells at DIV5 and DIV26. Cells were fixed in 4% paraformaldehyde in phosphate-buffered saline (4% PFA-PBS, Invitrogen) for 15 min at room temperature, permeabilized in 0.2% Triton X-100 (Sigma-Aldrich) for 5 min, rinsed in PBS and then washed twice with 0.15 M glycine in PBS for 5 min. Cells were preincubated in a blocking solution-PBS (Roche Applied Science, Mannheim, Germany) at room temperature for 30 min and then incubated with primary antibodies diluted in the blocking solution-PBS at room temperature for 1 hr. The primary antibodies used were polyclonal rabbit anti-MAP2 (1∶500; Covance, Harrogate, UK), anti-GFAP (1∶1000; Millipore, Billerica, MA, USA) and monoclonal mouse anti-NF-200 (1∶400; Sigma-Aldrich), anti-GFAP (1∶500; Millipore) or monoclonal mouse anti-GFAP Alexa-fluor 488 conjugated (1∶1000; Millipore) and anti-Nestin (1∶200; Millipore). The AMPA receptor subunit analysis was carried out with polyclonal rabbit anti-GluR1 (1∶40; Millipore), anti-GluR2 (1∶200; Millipore), anti-GluR3 (1∶50, Alomone Labs Ltd.), polyclonal goat anti-GluR4 (1∶50; Abcam, Cambridge, UK). Unconjugated primary antibodies were recognized by secondary antibodies conjugated with Alexa-fluor 488 or Alexa-fluor 555 dyes (Invitrogen). After exposure to each antibody, the cells were washed for at least 20 min in three changes of PBS and then mounted on coverslips in SlowFade Gold antifade reagent (Invitrogen). Images were captured on a Zeiss Axioplan2 microscope.

### Western blot analysis

Cells harvested from primary cortical cultures were solubilized with modified RIPA (50 mM Tris-HCl, pH 7.4, 150 mm NaCl, 1 mm EDTA, 1% IGEPAL CA630, 0.25% NaDOC, 0.1% SDS, 2% CHAPS and 1× protease inhibitor tablets; Roche Applied Science) and then sonicated. A portion of the lysate was used for the BCA protein concentration assay (Sigma-Aldrich). Before electrophoresis, each sample was incubated at 70°C for 10 minutes. Equal amounts of protein were applied to precast SDS polyacrylamide gels (4–12% NuPAGE Bis-Tris gels; Invitrogen) and the proteins were electrophoretically transferred to a Hybond-P PVDF Transfer Membrane (GE Healthcare, Waukesha, WI, USA) for 2 h at 1 mA/cm^2^ membrane surface. The membranes were blocked for 60 min with 3% nonfat dry milk or 2% BSA in TBS-T (Tris-buffered saline with 0.1% Tween-20, Sigma-Aldrich) and then incubated overnight at 4°C in the blocking solution with the primary antibodies corresponding to either rabbit polyclonal anti-GluR1 (1∶200, Millipore), rabbit polyclonal anti-GluR2 (1∶2500, Millipore), rabbit polyclonal anti-GluR3 (1∶500, Alomone Labs Ltd) or rabbit polyclonal anti-GluR4 (1∶100, Millipore) with mouse monoclonal anti-GAPDH (1∶2500, Sigma-Aldrich) as a loading control. After 3 washes in TBS-T, the membranes were incubated for 1 h at room temperature with HRP- (Millipore) or AP-conjugated (Santa Cruz Biotechnology, Santa Cruz, CA, USA) secondary antibodies. After 3 washes in TBS-T, immunolabeled proteins were detected by incubation with Supersignal West Pico Chemiluminescent Substrate (Pierce, Rockford, IL, USA) or CDPstar (Roche Applied Science) blotting detection reagents and then exposed to imaging film. For each experiment, the protein of interest was identified by reference to pre-stained Novex Sharp Protein Standards (Invitrogen) loaded on the same gel. The intensity of the immunoreactive bands was analyzed with Image-Pro Plus. Data are presented as the ratio of the optical density of the band of the investigated protein to that of the GAPDH band and are expressed as a percentage of controls. Each treatment was carried out and analyzed in three independent primary culture dishes.

### Quantitative real-time PCR (TaqMan)

Total RNA from cultured neurons was extracted using TRIZOL reagent (Invitrogen) according to the manufacturer's instructions. RNA quantification and quality controls were performed using spectrophotometric analysis and the AGILENT Bioanalyzer 2100 lab-on-a-chip technology (AGILENT Technologies, Santa Clara, CA, USA).

Reverse transcription was carried out using Moloney murine leukemia virus-reverse transcriptase (MMLV-RT) provided by Invitrogen. Briefly, 2 µg of total RNA were mixed with 2.2 µl of 0.2 ng/µl random hexamer (Invitrogen), 10 µl of 5× buffer (Invitrogen), 10 µl of 2 mM dNTPs, 1 µl of 1 mM DTT (Invitrogen), 0.4 µl of 33 U/µl RNasin (Promega, Madison, WI, USA) and 2 µl MMLV-RT (200 u/µl) in a final volume of 50 µl. The reaction mixture was incubated at 37°C for 2 h, and then the enzyme was inactivated at 95°C for 10 min.

To analyze the RNA expression pattern of glutamate AMPA receptors, we used the Applied Biosystems 7500 Real-time PCR system (Foster City, CA, USA) with Taqman probes following the manufacturer's instructions. 20 ng of sample were used in each real-time PCR (TaqMan Gene Expression Assay ID probes: GluR1: Rn00709588_m1; GluR2: Rn00568514_m1; GluR3: Rn00583547_m1; GluR4: Rn00568544 _m1; Applied Biosystems). The expression ratio of the target genes was calculated as described [61], using the geometric mean of the β-actin, GAPDH and Rplp2 transcripts (ID: β-actin: Rn00667869_m1; GAPDH: Rn99999916_s1; Rplp2: Rn03302266_gH) as a reference.

### Flip/Flop alternative splicing and editing quantification

The levels of the flip/flop splicing variants were evaluated by sequence analysis as described previously [62]. For each of the AMPA receptor subunits, both isoforms were amplified using a pair of common primers. The amplification products, consisting of both flip and flop isoforms at different concentrations were sequenced; the flip and flop exons appeared as peaks superimposed in the electropherogram. Flip and flop exons exhibit mismatches of A/G in specific locations. The relative expression level of the flip exon can thus be reliably calculated as the ratio of the peak area of G (flip) and the sum of the peak areas A+G in different positions. The areas representing the amount of each nucleotide were quantified using Discovery Studio (DS) Gene 1.5 (Accelrys Inc., San Diego, CA, USA), and the means and standard errors for each experimental group were calculated and used for subsequent statistical analysis. Quantification of the editing levels in the AMPA receptor GluR2, GluR3 and GluR4 transcripts was done by sequence analysis as previously described [63].

### Statistical analysis

Each experiment was performed in 3 independent preparations of rat cortical cultures. Statistical analysis of the editing and splicing data, mRNA and protein levels was performed using one-way ANOVA followed by specific *post-hoc* tests, as indicated in the figure legends.

## Supporting Information

Figure S1 **Global effect of chronic treatments on AMPA receptor mRNA variants.** 24 hour treatments with KCl, glutamate or APV/TTX affect the percentage composition of each single AMPA receptor mRNA isoform deriving from alternative splicing and R/G editing. GluR1 subunit is not subjected to RNA editing and carries an adenine thus coding for an arginine in the corresponding site, so that it is composed only by flip and flop variants. On the other hand, GluR2-4 are expressed as four different variants: R/G-unedited flip (A-flip), R/G-edited flip (G-flip), R/G-unedited flop (A-flop) and R/G-edited flop (G-flop).
(TIF)
